# Clinicopathological and EBV analysis of respiratory epithelial adenomatoid hamartoma

**DOI:** 10.1186/1746-1596-9-70

**Published:** 2014-03-25

**Authors:** Xing Hua, Xiaoxiao Huang, Zexiao Liao, Qi Xian, Lina Yu

**Affiliations:** 1 Department of Pathology, The Forth Affiliated Hospital of Jinan University, 396# Tong Fu Zhong Rd, Guangzhou 510515, China; 2 Department of Pathology, Nanfang Hospital, Southern Medical University, 510515 Tonghe, Guangzhou, People’s Republic of China; 3 Department of Pathology, College of Basic Medicine, Southern Medical University, 510515 Tonghe, Guangzhou, People’s Republic of China

**Keywords:** Respiratory epithelial adenomatoid hamartoma, Nasopharynx, Sinonasal tract, Epstein-Barr virus

## Abstract

**Background:**

To investigate the clinicopathological characteristics of respiratory epithelial adenomatoid hamartoma (REAH) in residents of Southern China and to study the correlation between REAH and Epstein-Barr virus (EBV).

**Methods:**

Clinicopathological data of 53 cases of REAH were retrospectively analyzed. The immunoreactivity for CK 7, CK20, CEA, p53, and Ki-67, Alcian blue–periodic acid-Schiff (AB-PAS) staining and in situ hybridization for EBV-encoded RNA (EBER) were carried out.

**Results:**

REAH lesions were covered with ciliated columnar epithelium and proliferation of subepithelial glands, which were positive for CK7, and negative for CK20, CEA, and p53. Goblet cell metaplasia was stained blue by AB-PAS. The frequency of EBER positive cases in REAH located in nasopharynx was 27.78%, compared with that in the nasal cavity (15.79%) and paranasal sinuses (12.50%), there were no statistical differences.

**Conclusions:**

REAH is an uncommon entity with distinctive morphologic features and EBV may have nothing to do with REAH.

**Virtual slides:**

The virtual slide(s) for this article can be found here:

http://www.diagnosticpathology.diagnomx.eu/vs/5875687401178748

## Introduction

The term "hamartoma" was introduced by Albrecht in 1904 to distinguish between true neoplasms and tumor-like lesions [1]. In 1934, Goldsworthy applied this term to a benign tumor in the lung composed predominantly of a combination of fat and cartilage [2]. This term is now used to designate a focal overgrowth of mature normal cells and tissues at sites of identical cellular composition; thus, a hamartoma may occur in any organ. It may arise from any of the germ layers and does not metastasize. Although hamartomas have been described as occurring throughout the body, they are rare in the upper aerodigestive tract [3]. Respiratory epithelial adenomatoid hamartoma (REAH) was first described in 1995 by Wenig and Heffner in a series of 31 cases [4]. REAH is a benign entity characterized by an abnormal proliferation of glandular tissue surrounded by a thick eosinophilic basement membrane within ciliated respiratory epithelium with no evidence of atypical or metaplastic changes in the squamous cells.

To date, there is limited literature regarding REAH [5]. In particular, no studies of REAH have been carried out in Cantonese individuals living in the central region of Guangdong Province in Southern China. Nasopharyngeal carcinoma (NPC) is common among the Cantonese, and Epstein-Barr virus (EBV) has been considered crucial for NPC cloning [6]. In the current study, we analyzed the clinicopathological characteristics of REAH in residents of Southern China and the correlation between EBV and REAH.

## Materials and methods

### Ethics statement

This study was carried out according to the principles of the Declaration of Helsinki and approved by the Institutional Review Board of the Fourth Affiliated Hospital of Jinan University and Nanfang Hospital of Southern Medical University. All patients provided written informed consent for the collection of samples and subsequent analysis.

The medical records of all 53 patients who were diagnosed with REAH between January 1998 and May 2011 were analyzed. Demographic and clinical data were obtained retrospectively from their hospital medical records, and paraffin-embedded tissue samples and slides were obtained from the Department of Pathology at the Fourth Affiliated Hospital of Jinan University and Nanfang Hospital of Southern Medical University in Guangzhou, China. All patients data and samples were anonymized before use. All of the slides were independently reviewed by five experienced pathologists to reach a diagnosis by consensus.

### Immunohistochemistry and AB-PAS histochemical staining

Ten sections, each 4 μm in thickness, were cut from the paraffin blocks of the REAH lesions. Consecutive sections were used to make an optimal comparison between morphology and protein expression. Immunohistochemical staining for cytokeratin (CK) 7, carcinoembryonic antigen (CEA), 34βE12, p63, p53, and Ki-67 was carried out as described elsewhere [7]. Primary antibodies against CK7 (clone OV-TL 12/30, 1:100 dilution), CEA (clone Ks20.8, 1:50 dilution), Collagen IV(clone PHM-12, 1:100 dilution), p63 (clone 4A4, 1:200 dilution), p53 (clone DO-7, 1:100 dilution), and Ki-67 (clone MIB-1, 1:25 dilution) were obtained from Dako Corporation (Glostrop, Denmark). Appropriate positive and negative controls were run in each case. As negative staining controls, the primary antibodies were replaced with the primary antibody diluents.

Alcian blue–periodic acid-Schiff (AB-PAS) staining was carried out by immersing each section in a solution of alcian blue and 3% acetic acid for 30 min, then in Schiff’s reagent for 15 min to promote oxidation, and then counterstaining it in hematoxylin.

### In situ hybridization for EBER

To determine the correlation between EBV and REAH, we divided our tissue samples into 4 groups: Group 1 consisted of normal tissue from the nasal cavity and paranasal sinuses mucosa (31 samples); group 2 consisted of REAH tissue taken from the same structures (31 samples); group 3 consisted of normal mucosal tissue from the nasopharynx (22 samples); and group 4 consisted of REAH tissue taken from the nasopharynx (22 samples).

In situ hybridization was performed using a flourescein-conjugated EBV peptide nucleic acid (PNA) probe (Dako) for EBV-encoded RNA (EBER) according to the manufacturer’s instructions. After the sections were dewaxed, they were rehydrated in graded alcohol and distilled water and predigested with proteinase K. A hybridization solution containing the fluorescein-conjugated EBV nucleic acid probe was then applied, followed by the application of alkaline-phosphatase–conjugated antibody to fluorescein isothiocyanate. Bromochloroindolyl phosphate/nitro blue tetrazolium chloride (BCIP/NBT) combined with levamisole was applied to serve as the chromogen. The slides were then lightly counterstained with hematoxylin. Dark blue or black staining of the nucleus served as a signal of hybridization. Positive and negative control slides were run for each specimen by replacing the EBV probe with a positive or negative fluorescein-conjugated PNA probe (Dako).

### Statistical analysis

The staining results of EBERs in REAH tissues were analyzed using Fisher’s exact test in comparison with that of control group obtained from corresponding normal tissue. *p* value of less than 0.05 was considered significant.

## Results

A total of 53 patients completed the study. They included 25 women and 28 men with an average age of 48.12 years (range: 17–64 years). Patient demographics are summarized in Table 1. Of these patients, 20.76% (n = 11) had surgery for chronic sinusitis, 45.28% (n = 24) had surgery for polyps, and 33.96% (n = 18) had a biopsy for suspicion of NPC.

**Table 1:** Patient demographics

**Characteristics**	**Number of cases**
Gender	
Male	28
Female	25
Age, year	
≤50	37
>50	16
Lesion location	
Nasal cavity	19
Paranasal sinuses	16
Nasopharynx	18
Main symptoms	
Nasal stuffiness	38
Deviated septum	11
Epistaxis	2
Rhinorrhea	14
Chronic recurrent sinusitis	27
Facial pain	29
Proptosis	1
Hyposmia	35
Recurrences	1

### Computed tomography

**Figure 1:**
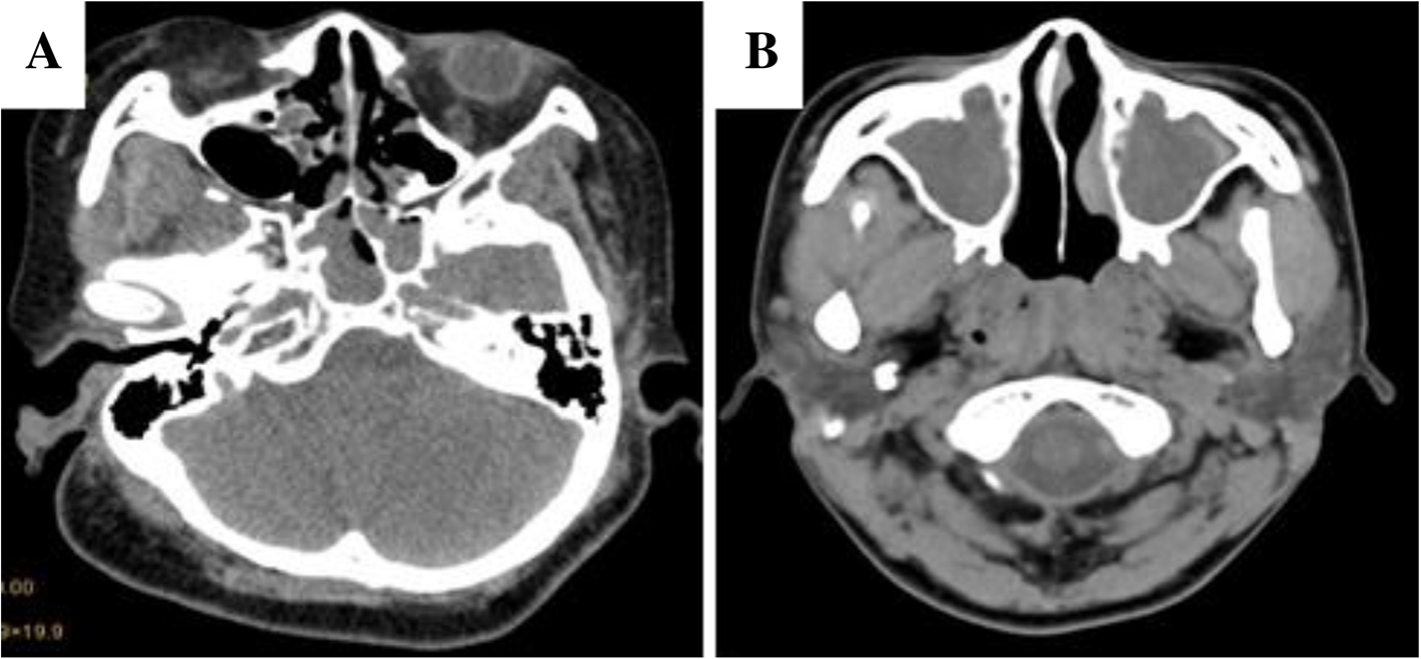
**Computed tomography scans of REAH. A.** Axial CT showing soft tissue masses in both nasal cavities, mucosa in maxillary sinus, ethmoid sinus thickening in some extent. **B.** Additional CT of maxilla. Additional CT Note occlusion of right posterior choana and nasopharynx by mass.

Preoperative computed tomography (CT) scans ordered by their primary care physicians revealed no distinguishing features that could be used to confirm REAH. CT scans showed soft tissue masses in the nasal cavity or paranasal sinuses and thickening (to some extent) of the mucosa in the maxillary sinus, ethmoid sinus, frontal sinus and sphenoid sinus. Radiographic imaging revealed mainly chronic sinusitis and nasal polyps (Figure 1A). Additional CT findings included bony irregularities in the lateral maxillary wall and orbital floor. In patients with REAH in the nasopharynx, CT scans revealed thickening of the nasopharyngeal wall, the disappearance or flattening of pharyngeal recesses, and filling of the nasopharyngeal cavity with soft tissue masses but no sign of destruction of craniobasilar bone or swelling of cervical lymph nodes (Figure 1B).

### Histologic features

**Figure 2:**
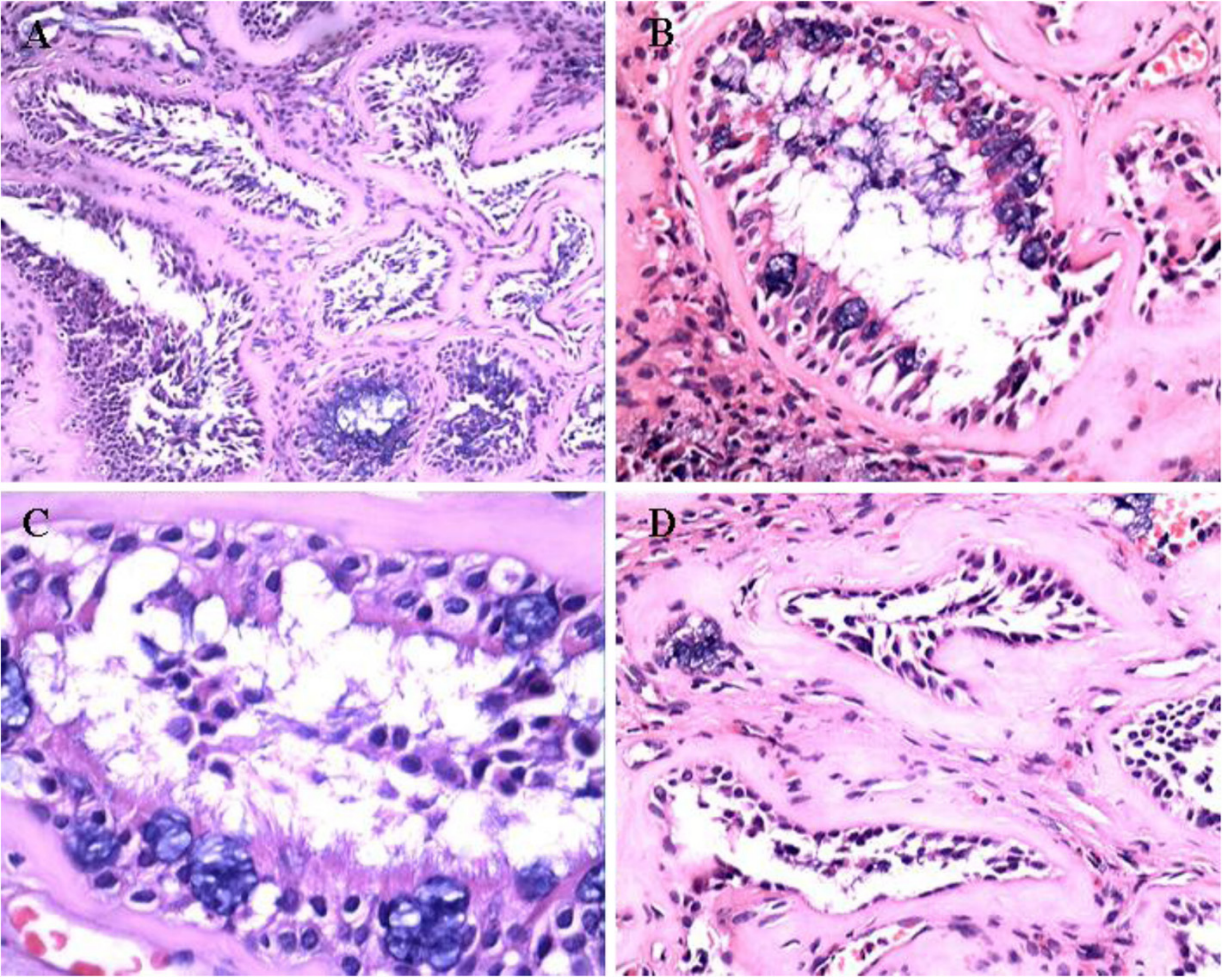
**Respiratory epithelial adenomatoid hamartoma. A.** The glands proliferated with various sizes (hematoxylin-eosin, 20×). **B.** The glands were lined by ciliated respiratory epithelium (hematoxylin-eosin, 20×). **C.** Most of the ciliated respiratory epithelial are metaplastic goblet cells (hematoxylin-eosin, 40×). **D.** The glands were surround by thick eosinophilic and hyalinizated basement membranes (hematoxylin-eosin, 20×).

A histopathological examination revealed that REAH lesions were covered with ciliated columnar epithelium and a proliferation of subepithelial glands. These glands were typically round to oval in shape and small to medium in size with a prominent dilation, unlike the cribriform glandular growth often seen in more aggressive tumors (Figure 2A). The glands were lined with cuboidal or flat ciliated epithelial cells (Figure 2B). Metaplasia was often observed in the mucinous glands, and most of the metaplastic cells were goblet cells (Figure 2C). A characteristic finding was stromal hyalinization with a thick eosinophilic basement membrane separating the glands (Figure 2D). Other histologic features included fibroblastic proliferation, stromal edema, and a mixed inflammatory cell infiltrate.

### Immunohistochemistry and histochemical staining

**Figure 3:**
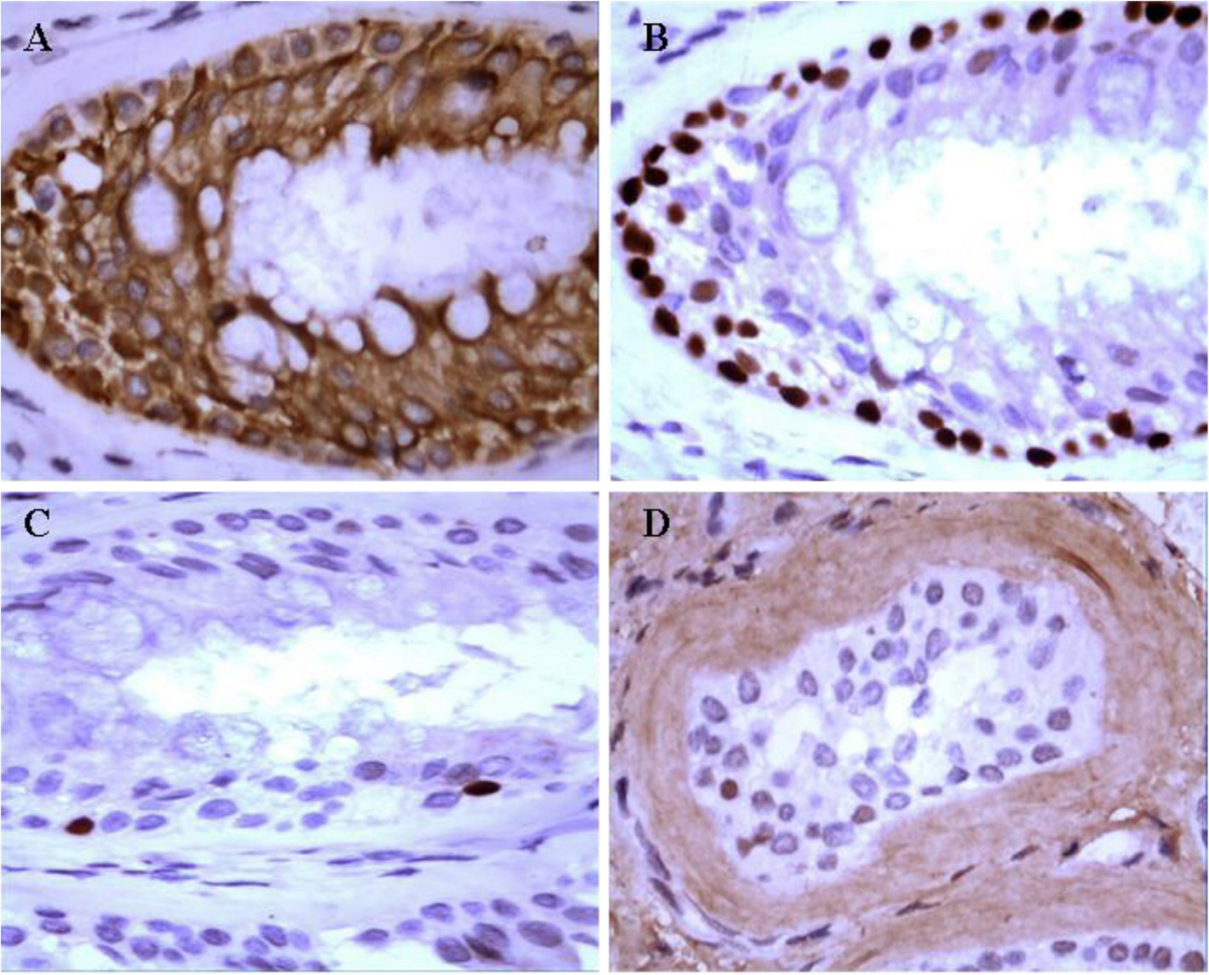
**Representative immunohistochemical staining results of REAH. A.** REAH stained with cytokeratin 7 shows diffuse predominantly strong staining of the glandular epithelium. **B.** p63 outlining the basal cell layers around glands in REAH. **C.** Ki-67 staining was localized to the basal layer with a labeling index less than 1%. **D.** The distinctive hyalinized basement membrane that surrounds the ciliated epithelium was highlighted by Collagen IV.

**Figure 4:**
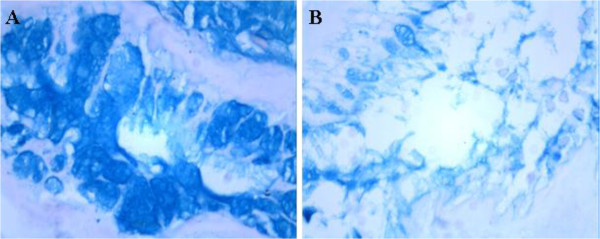
**Alcian blue–periodic acid–Schiff Histochemical Staining.** Compared with the respiratory epithelium of normal tissue from the nasal cavity mucosa **(B)**, more mucins took up the blue AB-PAS stain in the glandular epithelium of REAH **(A)**.

Stromal epithelial components of REAH tissue were positive for CK7 (Figure 3A) and negative for CK20. Both epithelial components and basal cells were negative for p53. p63 outlining the basal cell layers around glands inREAH (Figure 3B) and Ki-67 staining was only localized to the basal layer, where it indicated the lowest mean labeling index of any group of lesions (<1%) (Figure 3C). The distinctive hyalinized basement membrane that surrounds the ciliated epithelium was positive for collagen IV (Figure 3D). Compared with that of normal ciliated respiratory epithelium, more mucins took up the blue AB-PAS stain in the glandular epithelium of REAH (Figure 4).

### EBER in situ hybridization

**Figure 5:**
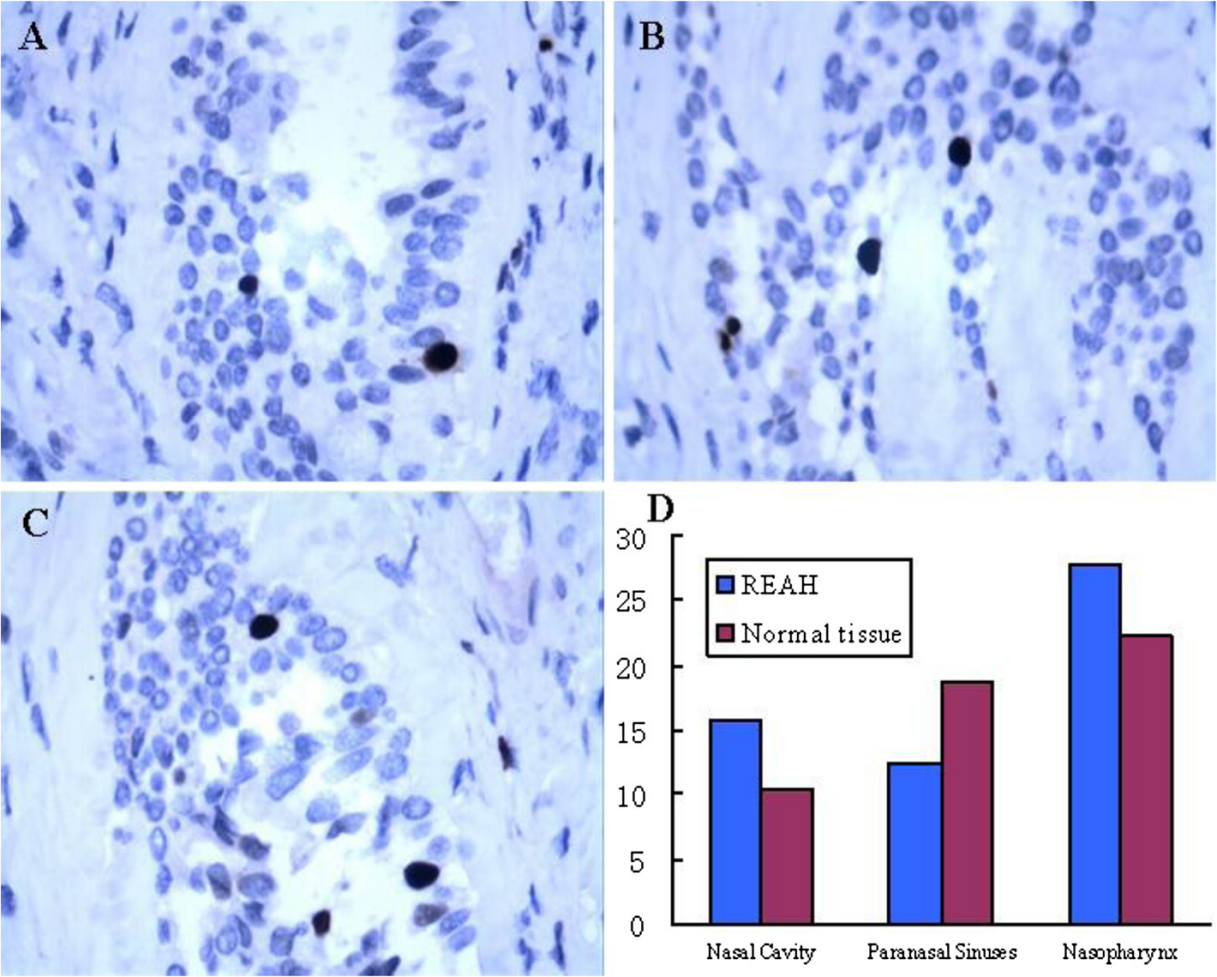
**Epstein-Barr virus–encoded RNA (EBER) in-situ hybridization (ISH) of REAH.** Black/dark brown signal is localized to the nuclei of affected cells. **A** (nasal cavity), **B** (paranasal sinuses) and **C** (nasopharynx) showing REAH with rare positive cell for EBER. **D.** Results of EBER positive rate compared with that of corresponding normal tissue.

In situ hybridization was used to detect the expression of EBER in REAH tissue. As shown in Figure 5, EBER was found sporadically positive expressed in some of the obtained tissues and black/dark brown signal was localized to the nuclei of affected cells. As shown in Table 2, among the various locations from which REAH tissue was obtained, EBV was found sporadically in REAH epithelial cells in 15.79% (3/19) of tissue samples obtained from the nasal cavity, 12.50% (2/16) of tissue from the paranasal sinuses, and 27.78% (5/18) of tissue from the nasopharynx. Although EBER was found most frequently in the nasopharynx, the difference in frequency in the nasopharynx compared with the other sites was not statistically significant (*P* = 0.268). Additionally, 10.53% (2/19) of tissue samples obtained from the nasal cavity, 18.75% (3/16) of tissue samples from the paranasal sinuses, and 22.22% (4/18) of tissue samples from the nasopharynx were shown EBER sporadically positive (Figure 5). There was also no statistical difference in EBER expression between REAH and normal mucosal tissue.

**Table 2:** EBV-encoded small RNAs staining in REAH and corresponding normal tissues

**Lesion location**	**Positively stained for EBERs**		**p**^**a **^**value**
	**REAH**	**Normal tissue**	
Nasal cavity	3/19 (15.79%)	2/19 (10.53%)	0.523
Paranasal sinuses	2/16 (12.50%)	3/16 (18.75%)	0.527
Nasopharynx	5/18 (27.78%)	4/18 (22.22%)	0.530

^a^Derived from Fisher’s exact test as comparison with the control groups. EBERs = EBV-encoded small RNAs.

## Discussion

REAH is an expansile mass that causes upper respiratory symptoms and discomfort mainly in adults [5]. Symptoms at presentation vary and are similar to those that accompany chronic sinusitis, eg, nasal congestion, nasal obstruction, headaches, facial pain, epistaxis, and hyposmia. Endoscopy does not reveal any distinguishing features to suggest a diagnosis of REAH, and neither CT nor magnetic resonance imaging produces a specific signal intensity that can help the clinician distinguish REAH from other sinus lesions [3,8,9]. Distinctive histologic features of REAH include a glandular component that originates in the overlying surface respiratory epithelium and polypoid growths that represent a proliferation of respiratory epithelial adenomatoid tissue [5]. It is important to recognize this lesion, however, because it can be confused histopathologically with other disease processes that require a significantly different treatment approach. Included in the microscopic differential diagnosis of REAH are inflammatory polyps [4,9,10], inverted Schneiderian papillomas [3,4,10,11], and well-differentiated adenocarcinoma [3,11]. Pathologists must be aware of this entity to avoid overdiagnosis and overly aggressive surgical procedures.

Mucins are conventionally classified as neutral mucins or acid mucins (the latter comprising sulfomucins and sialomucins) according to the color reaction obtained with histochemical stains such as periodic acid–Schiff (PAS) and Alcian blue (AB) at pH 2.5 [12]. Airway mucins are produced by two different types of cells: goblet cells in the surface epithelium and mucous cells in submucosal glands. In our study, more mucins took up the blue AB-PAS stain in REAH tissue than in controls. This suggests that REAH-associated metaplasia develops predominantly in the goblet cells in ciliated respiratory epithelial tissue and that acid mucin production is increased in the airways of patients with this disease.

Mucus protects the underlying airway epithelium from dehydration and from damage by pathogens or chemical and particulate irritants [13,14]. It has been proposed that an abnormal increase in mucus secretion promotes bacterial adhesion and inhibits bacterial clearance by impeding cilial function [15,16], which may exacerbate symptoms in patients with REAH.

REAH is frequently associated with inflammatory polyps, although it has been suggested that these polyps arise secondary to the inflammatory process. The mechanisms responsible for inducing REAH are still unknown.

To date, there has been only one study of the molecular genetics of REAH. Ozolek and Hunt showed that patients with REAH lack heterozygosity for loci on chromosomes 9p and 18q and exhibit a fractional allelic loss of 31%, which the authors concluded was unusually high for a nonneoplastic entity. They suggested that REAH may, in fact, be a benign neoplasm and not a hamartoma, as was originally believed [17]. In our study, the nuclear features of REAH were bland and both epithelial components and basal cells were negative for p53. Ki-67 staining was only localized to the basal layer and the labeling index less than 1%. These findings support the benign nature of the lesion.

REAH is a rare lesion that is limited to the nasal cavity, paranasal sinuses, and nasopharynx. Wenig and Heffner estimated that approximately 70% of REAH tissue is in the nasal cavity and 9.68% is in the nasopharynx [4]. However, our data, suggest that 35.85% of REAH tissue is in the nasal cavity, 30.19% in the paranasanl sinuses and 33.96% in the nasopharynx.

NPC is a nonlymphomatous squamous-cell carcinoma that arises in the epithelial lining of the nasopharynx. This neoplasm is uncommon in most countries, with an age-adjusted incidence in both sexes of fewer than 1:100,000. It occurs with much greater frequency in Southern China, however, especially among Chinese people living in the province of Guangdong (25 to 30:100,000 annually) [18-20]. In contrast to other head and neck cancers and to epithelial malignancy in general, NPC is unique in terms of its strong association with EBV, which is consistently detected in patients with NPC, whether they are from regions with a high or low incidence of NPC [21,22].

The EBER signal has been shown through in situ hybridization to be present in nearly all tumor cells. In NPC cells, EBV exists as an episome and is not integrated into the host genome. EBV adopts a specific form of latent infection, latency II, in NPC cells. Only a limited number of viral gene transcripts—including transcripts for *EBER*, *EBNA1, LMP1, LMP2, BARF1,* and several *BamHI*s—are expressed. A latent EBV infection may take place before expansion of the malignant cell, which would indicate that EBV plays a critical role in transforming nasopharyngeal epithelial cells into an invasive form of cancer [22-24].

In our study, all of the patients were from Guangdong, and most of their REAH lesions were found in the nasopharynx. To the best of our knowledge, ours was the first attempt to study the correlation between EBV and REAH in Southern China.

In situ hybridization has been proven to be a valuable molecular tool for the diagnosis and understanding of viral and neoplastic disease [25,26]. This test provides optimal DNA detection sensitivity of approximately 40 kb in tissue sections and 10 to 20 copies of mRNA or viral DNA per cell. It is more sensitive to EBER than polymerase chain reaction analysis in the search for EBV latency in tissues and allows for localization of the EBV signal in tumor cells. In situ hybridization of EBER was reported to have a sensitivity of 98% and a specificity of 100% in detecting primary NPC.

The cancer-associated herpesvirus EBV infects more than 90% of the world’s population. Initial infection of EBV in human is usually asymptom or causes mononucleosis syndrome. Several neoplasms have been shown to be associated with EBV as evidenced by the presence of EBV genome in the tumor cells [27]. In our study, EBER was found sporadically positive expressed in some of the obtained tissues. Despite the fact that EBER was detected more frequently in REAH tissue, we found no statistically significant difference in EBV latency among tissues obtained from the nasal cavity, paranasal sinuses, and nasopharynx of patients with REAH or between REAH and normal mucosal tissue. We concluded that EBV may have nothing to do with REAH. These observations lend support the suggestion that REAH is a reactive rather than neoplastic lesion. Further investigation through molecular studies will be essential to further our understanding of the pathogenesis of this disease.

## Competing interests

The authors declare that they have no competing interests.

## Authors’ contributions

XH and LNY participated in the design of the study and wrote the manuscript. XXH, ZXL and QX carried out the H&E and IHC staining. XH and LNY collected the clinical data and reviewed H&E and IHC slides. ZXL performed the statistical analysis. All authors read and approved the final manuscript.
